# March hemoglobinuria progressed to acute kidney injury after kendo practice: a case report

**DOI:** 10.1186/s12882-022-02988-0

**Published:** 2022-11-16

**Authors:** Maiko Yoshida, Hitoshi Suzuki, Sho Hamaguchi, Masako Iwasaki, Hiromitsu Fukuda, Hisatsugu Takahara, Shigeki Tomita, Yusuke Suzuki

**Affiliations:** 1 Department of Nephrology, Juntendo University Urayasu Hospital, 2-1-1 Tomioka, Urayasu-Shi, Chiba, 279-0021 Japan; 2 Department of Pathology, Juntendo University Urayasu Hospital, Chiba, Japan; 3 Department of Nephrology, Juntendo University Faculty of Medicine, Tokyo, Japan

**Keywords:** March hemoglobinuria, Exertional hemoglobinuria, Hemosiderin deposit, Renal biopsy, Acute kidney injury

## Abstract

**Background:**

March hemoglobinuria is caused by a hemolytic mechanism due to transient hematuria after physical exercise which, although rare, may lead to acute kidney injury. We report a case of a patient with march hemoglobinuria induced by kendo, which was diagnosed by the presence of Berlin blue iron staining in the proximal tubules through renal biopsy.

**Case presentation:**

A 15-year-old male complained of fever (37 °C), general malaise, and nausea after hard kendo sessions. Laboratory findings revealed indirect bilirubin dominant hyperbilirubinemia (total bilirubin 3.8 mg/dL), high lactate dehydrogenase (LDH), and acute kidney injury (serum creatinine: 3.11 mg/dL and estimated glomerular filtration rate: 26 mL/min/1.73m^2^). Urine test was positive for occult blood but without hematuria. Renal biopsy was performed to clarify the cause of renal injury, which showed minor glomerular abnormalities. Meanwhile, hemosiderin deposition was identified in the proximal tubules by Berlin blue iron staining, and lysosomes were observed to contain granular iron. In addition to clinical background of strenuous kendo exercise, renal biopsy led to a definitive diagnosis of march hemoglobinuria.

**Conclusions:**

March hemoglobinuria is a hemolytic disease that can occur after intense exercise, especially kendo. Considering its rarity due to the lack of critical symptoms, it is important to note that occult blood-positive findings may be indicative of march hemoglobinuria if the patient underwent strenuous exercise. Therefore, clinicians should be aware of this possibility to provide timely and appropriate treatment.

## Background

March hemoglobinuria is a hemolytic disease characterized by transient hematuria after strenuous exercise, especially long-walking and running; it was first recognized by Fleisher in 1881 [1]. In addition to the exercise distance and speed, the hardness of the road surface and properties of the shoes worn were shown to greatly influence the onset of march hemoglobinuria. In Japan, kendo, a martial art, is one of the triggers of march hemoglobinuria [2]. Gross hematuria triggered by kendo is caused by physical and mechanical intravascular hemolysis in the capillaries on the sole of the foot as a result of stepping exercises on bare feet. Since macroscopic hematuria disappears shortly after the end of exercise, it is often left unattended without visiting a hospital. Major symptoms associated with hemoglobinuria include nausea, abdominal pain, myalgia, and foot pain [3].

Cases of march hemoglobinuria accompanied by acute kidney injury are very rare [4]. Thus, we report a case of march hemoglobinuria induced by kendo in which Berlin blue iron staining was observed in proximal tubular cells obtained via renal biopsy.

## Case presentation

A healthy 15-year-old Japanese male presented with fever (37°C), general malaise, and nausea after intense kendo exercise for 2 days. Laboratory findings revealed hyperbilirubinemia (total bilirubin 3.8 mg/dL), acute kidney injury (serum creatinine: 3.11 mg/dL, and an estimated glomerular filtration rate (eGFR) of 26 mL/min/1.73m^2^). The levels of serum creatine kinase was within normal limit. Thus, it is suggested that the cause of hemoglobinuria is hemolysis, but not rhabdomyolysis. It was no metabolic acidosis and electrolyte abnormalities. He had no history of kidney disease and was not taking any medications. He had hyperuricemia (uric acid 12.7 mg/dL), hyperbilirubinemia (indirect bilirubin dominant), high lactate dehydrogenase (LDH), and his urine test indicated positive results for occult blood; however, hematuria was not detected (Table 1). At the time of the renal biopsy, the patient’s height, weight, the percentiles of the length and the body weight , heart rate and blood pressure were 173.5 cm, 64.6 kg, 71.57 percentile and 72 percentile, 53/min and 144/83 mmHg, respectively. Physical examination findings were normal. Abdominal computed tomography (CT) revealed no abnormalities in both kidneys. We performed renal biopsy for a definitive diagnosis.

**Table 1:** Clinical data at the time point of renal biopsy

Parameter	Value (reference range)	Parameter	Value (reference range)	Parameter	Value (reference range)
**Hematological**		**Blood biochemistry**		**Immunological test**	
White blood cell count, /μL	8100 (4000-8000)	Total protein, g/dL	7.0 (6.7-8.3)	CH50, IU/mL	77 (32-49)
Hemoglobin, g/dL	15.6 (14-18)	Albumin, g/dL	4.7 (3.9-4.9)	C3, mg/dL	128 (65-135)
Platelets, ×10^4^/μL	14.1 (15-35)	Blood urea nitrogen, mg/dL	30 (8-22)	C4, mg/dL	28 (13-35)
Reticulocyte, %	1.3 (0.2-2.8)	Creatinine, mg/dL	2.98 (0.61-1.04)	IgG, mg/dL	867 (870-1700)
		eGFR, mL/min/1.73m^2^	27 (>60)	IgA, mg/dL	197 (110-410)
**Coagulation test**		Cystatin C, mg/dL	1.35 (0.63-0.95)	IgM, mg/dL	56 (35-220)
within normal range		Uric acid, mg/dL	12.7 (2-7)		
		Creatinine kinase, mg/dL	127 (60-287)	Antinuclear antibody, IU/mL	<40 (<40)
**Urinalysis**		Sodium, mmol/L	139 (138-146)	MPO-ANCA, IU/mL	<1.0 (0-3.5)
pH	5.0 (4.5-8.0)	Potassium, mmol/L	4.4 (3.6-4.9)	PR3-ANCA, IU/mL	<1.0 (0-3.5)
Specific gravity	1.011 (1.005-1.025)	Chloride, mmol/L	99 (99-109)	anti-GBM, U/mL	<2.0 (0-3)
Protein urea, g/g creatinine	<0.15 (<0.15)	Calcium, mg/dL	9.2 (8.7-10.3)	ASO, IU/mL	<10 (0-239)
Red Blood Cell, /HPF	1~4 (<5)	Glucose, mg/dL	98 (70-109)	ASK	<20 (<20)
White Blood Cell, /HPF	5~9 (<5)	Bilirubin totally, mg/dL	2.9 (0.2-1.3)	cryoglobulin	negative
U-NAG, IU/L	7.4 (0.7-11.2)	Bilirubin direct, mg/dL	0.9 (0-0.3)		
U-β2MG, μg/L	10 (0-230)	AST, IU/L	14 (13-33)	Haptoglobin, mg/dL	183 (19-170)
U-α1MG, mg/L	2.3 (1-15.5)	ALT, IU/L	11 (8-42)	DAT	negative
L-FABP, μg/g creatinine	0.81 (0-8.4)	LDH, IU/L	313 (124-222)		
		Total cholesterol, mg/dL	139 (128-219)		
**Blood gas analysis**		LDL cholesterol, mg/dL	82 (70-139)		
pH	7.313 (6-8)	Triglyceride, mg/dL	83 (30-149)		
HCO_3_, mmol/L	25.9 (0-99.9)	C-reactive protein, mg/dL	1.9 (0-0.3)		
Base excess, mmol/L	-1.1 (-99~99)	HbA1c, %	5.3 (4.6-6.2)		
**Hemolysis test**					
Ham test	negative				
erythrocyte fragility test	negative				

*U-NAG* Urinary N-acetyl-β-D-Glucosaminidase, *U-β2MG* Urinary beta2-microglobulin, *U-α1MG* Urinary alpha1-microglobulin, *eGFR* Estimated glomerular filtration rate, *L-FABP*, L-type fatty acid-binding protein, *AST* Aspartate amino transferase, *ALT* Alanine aminotransferase, *LDH* Lactate dehydrogenase, *LDL* Low density lipoprotein, *HbA1c* Hemoglobin A1c, *Ig* Immunoglobulin, *MPO* Myeloperoxidase, *PR3* Proteinase, *ANCA* Anti-neutrophil cytoplasmic antibody, *GBM* Glomerular basement membrane, *ASO* Anti-streptolysin, *ASK* Anti-streptokinase antibody, DAT Direct antiglobulin test

**Fig. 1:**
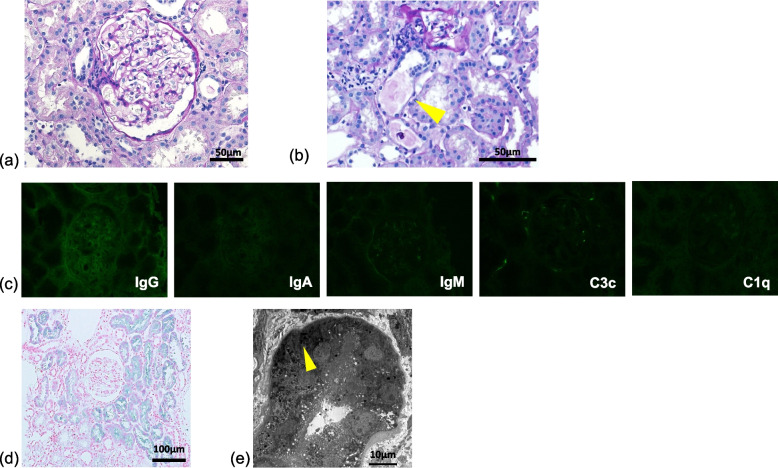
Light microscopy Olympus BX53, OLYMPUS DP73 camera, and cellSense (Olympus Corporation, Center Valley, PA, USA) wass used to capture the images. The images were obtained with eyepiece at 10X magnification and objective at 20X and 40X. No enhancement of the images was performed. The measured resolution was 4800 × 3600. **a** Histological findings show minor glomerular abnormalities (periodic acid–Schiff, 400 ×). **b** There is cast and hemosiderin deposition in the tubules (periodic acid–Schiff, 400 ×). **c** There are no significant findings in the immunofluorescence analysis. **d** Massive hemosiderin deposition detected in the proximal tubules by Berlin blue iron staining (200 ×). **e** Electron dense deposits was not detected by electron microscopy; however, lysosomes incorporated with iron granules are observed in the proximal tubules

Histological findings showed minor glomerular abnormalities (Fig. 1a). We observed cast and hemosiderin deposition in the tubules (Fig. 1b). There were no significant findings in immunofluorescence analysis (Fig. 1c). Massive hemosiderin deposition was detected in the proximal tubules by Berlin blue iron staining (Fig. 1d). Electron dense deposits were not detected by electron microscopy; however, lysosome incorporated iron granules were observed in the proximal tubules (Fig. 1e). Renal biopsy certified hemosiderin deposition in the proximal tubules, suggestive of march hemoglobinuria.

**Fig. 2:**
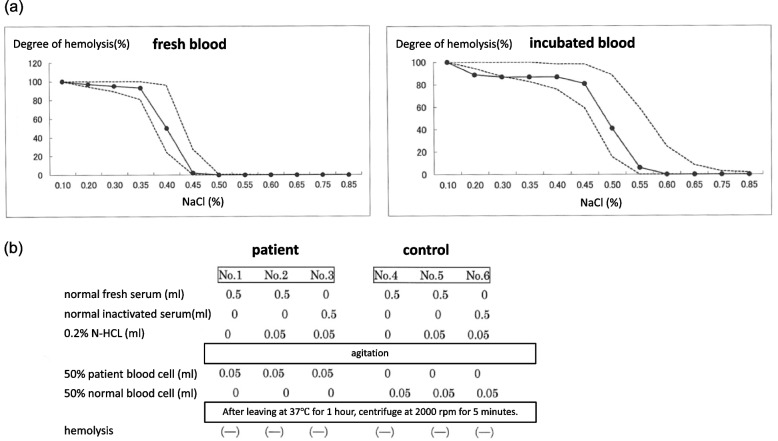
**a** The osmotic fragility test. The inside of the broken line is hemolysis in the normal range, and the solid line shows the degree of hemolysis of this patient. The patient’s red blood cells showed a degree of hemolysis within the normal range in both fresh and incubated red blood cells. **b** The test tubes of No. 1 to  3 were patient's group with patient's red blood cells and No. 4 to 6 test tubes were control group with normal red blood cells. Paroxysmal nocturnal hemoglobinuria (PNH) shows hemolysis with a mixture of acidified serum and patient erythrocytes (the No.2 test tube is positive), but inactivated serum and patient erythrocytes do not hemolyze even with acidification (the No. 3 test tube is negative). Present case showed negative response in all No. 1 to 3 test tubes

The patient was advised to rest and underwent fluid replacement with 1000 ml of hypotonic solution contained 90 mmol/L of sodium from day 1 to day 3 during hospitalization in addition to whole hospital diet and 1000ml of oral rehydration solution with electrolyte. The output of each day was about 1500-2000ml/day, thus leading to a normal kidney function (eGFR was improved from 27 to 90 mL/min/1.73m^2^) and bilirubin level. Hyperbilirubinemia and occult blood in the urine test were due to hemolysis. We also examined red blood cell fragility; however, the osmotic fragility test of erythrocytes and Ham’s test were negative (Fig. 2a,b). This patient did not show any abnormalities in numbers of erythrocyte as well as peripheral smear. Moreover, hyperbilirubinemia and occult blood in the urine were improved after resting and hydration. Thus, we did not measure glucose-6-phosphate dehydrogenase (G6PD) in present case. We have followed up 1 year. This patient has resumed kendo practice, however it has been stable without any acute renal injury and hemolysis due to avoiding dehydration and hard exercise.

## Discussion and conclusions

March hemoglobinuria is a disease that presents with transient hemoglobinuria after strenuous exercise, such as a marathon, karate, and kendo. Kendo is a form of Japanese fencing characterized by repetitive stepping and is thought to result in intravascular hemolysis due to mechanical stress. Aspartate aminotransferase, creatine kinase, and lactate dehydrogenase (LDH) increased, serum haptoglobin decreased, and urinary occult blood was positive immediately after a stress test in kendo [5]. March hemoglobinuria developed not only with foot but also hand hemolysis, which was caused by playing the conga drum [6]. In the present case, hemolytic findings, such as an indirect predominant increase in bilirubin and an increase in LDH, were observed. If the urinary occult blood is positive but urinary red blood cells are not found, hemoglobinuria and myoglobinuria need to be differentiated, and paroxysmal nocturnal hemoglobinuria (PNH), rhabdomyolysis, and march hemoglobinuria should be considered.

When intravascular hemolysis persists, free hemoglobin released into the plasma by hemolysis strongly adsorbs nitric oxide, which has smooth muscle relaxation and platelet aggregation inhibition actions, resulting in vascular spasm and digestion. The function of vascular endothelial cells is often impaired, causing intravascular thrombosis [7]. In patients with PNH, 65% of the population had chronic kidney disease (CKD) and 21% were in CKD stages 3–5 [8]. It is believed that the mechanism of renal dysfunction caused by intravascular hemolysis is due to the deposition of progressive hemosiderin in the proximal tubules, which was not filtered because of its high molecular weight. Hemosiderin is thought to accumulate in the epithelium, causing tubular atrophy and interstitial fibrosis, leading to renal injury [9]. Case reports of acute kidney injury caused by march hemoglobinuria are rare because the volume of hemolyzed blood is small compared to PNH. Gilligan DR, et al. reported that the volume of hemolysis is only 6–40 mL in patients with march hemoglobinuria [10]. There were no abnormalities in urinary levels of NAG, β2MG, α1MG and L-FABP in present case (Table 1). Previous report also indicated no tubular dysfunction [11]. Thus, there are very few case reports of renal biopsy for march hemoglobinuria [11–13]. In the present case, hemosiderin deposition was observed in the proximal tubule, and iron granules were also taken up in lysosomes, as observed in electron microscopic findings, which led to a definitive diagnosis of march hemoglobinuria.

It is also noteworthy that the cause of march hemoglobinuria may be an abnormality in the erythrocyte membrane. Banga JP, et al. found absent low-molecular-weight protein bands of the erythrocyte membrane structure in three cases of march hemoglobinuria [14]. Although the mechanism that explained the defect causing hemolysis has not yet been elucidated, march hemoglobinuria may be induced when patients with erythrocyte membrane anomalies exercise. However, no abnormalities in the erythrocyte membrane were detected in present case. This patient did not show any abnormalities in numbers of erythrocyte as well as peripheral smear. Moreover, hyperbilirubinemia and occult blood in the urine were improved after resting and hydration. Thus, we did not measure G6PD in present case. Even in patients with no abnormalities in the erythrocyte membrane, exercise itself has the potential to cause changes in the erythrocyte fragility and impaired membrane structure, eventually promote to hemolysis [15]. Platt OS, et al. proved that dehydrated cells were more sensitive to shear stress and were turned to fragile [16]. It is suggested that severe dehydration during hard exercise enhanced intravascular hemolysis and tubular atrophy due to accumulation of hemosiderin, leading to acute kidney injuries.

It could be possible to prevent the recurrence of hemoglobinuria by wearing shoes [17], which would function to defend the feet from external stimuli. It was previously reported that hemoglobinuria after exercise caused by karate disappeared through the use of a sponge rubber cushion [18]. March hemoglobinuria is a hemolytic disease that occurs after intense exercise, and its prevalence is very rare due to a lack of a definitive diagnosis. However, it is beneficial for patients to receive lifestyle guidance via a definitive diagnosis.

In conclusion, this report serves as a reminder that march hemoglobinuria can occur in occult blood-positive cases after intense exercise.

## Data Availability

The datasets used during the current study available from the corresponding author on reasonable request.

## Abbreviations

eGFR: Estimated glomerular filtration rate
LDH: Lactate dehydrogenase
CT: Computed tomography
CKD: Chronic kidney disease
G6PD: Glucose-6-phosphate dehydrogenase
NAG: N-acetyl-β-D-glucosaminidase
β2MG: Beta2-microglobulin
α1MG: Alpha1-microglobulin
L-FABP: L-type fatty acid-binding protein

## References

[1] Fleischer R (1881). Ueber eine neue Form von Haemoglbinuric beim menschen. Berl Klin Wschr.

[2] Shiraishi M (1989). Myoglobinuria and Kendo. Japanese J Clin Sports Med.

[3] Davidson RJ (1969). March or exertional haemoglobinuria. Semin Hematol.

[4] Pollard TD, Weiss IW (1970). Acute tubular necrosis in a patient with march hemoglobinuria. N Engl J Med.

[5] Urabe M, Hara Y, Hokama A, Suzuki M, Wakabayashi T, Ishii J (1986). A female case of march hemoglobinuria induced by kendo (Japanese fencing) exercise. Nippon Naika Gakkai Zasshi.

[6] Kaden WS (1970). Traumatic haemoglobinuria in conga-drum players. Lancet.

[7] Tracz MJ, Alam J, Nath KA (2007). Physiology and pathophysiology of heme: implications for kidney disease. J Am Soc Nephrol.

[8] Hillmen P, Elebute M, Kelly R, Urbano-Ispizua A, Hill A, Rother RP (2010). Long-term effect of the complement inhibitor eculizumab on kidney function in patients with paroxysmal nocturnal hemoglobinuria. Am J Hematol.

[9] Zhou XJ, Laszik Z, Wang XQ, Silva FG, Vaziri ND (2000). Association of renal injury with increased oxygen free radical activity and altered nitric oxide metabolism in chronic experimental hemosiderosis. Lab Invest.

[10] Gilligan DR, Altschule MD (1950). March hemoglobinuria in a woman. N Engl J Med.

[11] Nishioka R, Sofue T, Onishi K, Fujita T, Ozaki T (2017). A case of hemoglobinuria syndrome with kendo march with iron deposits in the renal tubules. Nippon Naika Gakkai Zasshi.

[12] Sugimoto K, Miyazawa T, Enya T, Nishi H, Miyazaki K, Okada M (2016). Clinical and genetic characteristics of Japanese nephronophthisis patients. Clin Exp Nephrol.

[13] Qian Q, Nath KA, Wu Y, Daoud TM, Sethi S (2010). Hemolysis and acute kidney failure. Am J Kidney Dis.

[14] Banga JP, Pinder JC, Gratzer WB, Linch DC, Huehns ER (1979). An erythrocyte membrane-protein anomaly in march haemoglobinuria. Lancet.

[15] Lippi G, Sanchis-Gomar F (2019). Epidemiological, biological and clinical update on exercise-induced hemolysis. Ann Transl Med.

[16] Platt OS, Lux SE, Nathan DG (1981). Exercise-induced hemolysis in xerocytosis. Erythrocyte dehydration and shear sensitivity. J Clin Invest..

[17] Davidson RJ (1964). Exertional Haemoglobinuria: A Report On Three Cases With Studies On The Haemolytic Mechanism. J Clin Pathol.

[18] Streeton JA (1967). Traumatic haemogloinuria caused by karate exercises. Lancet.

